# Transcription Factor Reb1p Regulates *DGK1*-encoded Diacylglycerol Kinase and Lipid Metabolism in Saccharomyces cerevisiae*

**DOI:** 10.1074/jbc.M113.507392

**Published:** 2013-08-22

**Authors:** Yixuan Qiu, Stylianos Fakas, Gil-Soo Han, Antonio Daniel Barbosa, Symeon Siniossoglou, George M. Carman

**Affiliations:** From the ‡ Department of Food Science, Rutgers Center for Lipid Research, and New Jersey Institute for Food, Nutrition, and Health, Rutgers University, New Brunswick, New Jersey 08901 and; the § Cambridge Institute for Medical Research, University of Cambridge, Cambridge CB2 0XY, United Kingdom

**Keywords:** Diacylglycerol, Glycerolipid, Phosphatidate, Phospholipid, Phospholipid Metabolism, Phospholipid Turnover, Diacylglycerol Kinase

## Abstract

In the yeast *Saccharomyces cerevisiae*, the *DGK1*-encoded diacylglycerol kinase catalyzes the CTP-dependent phosphorylation of diacylglycerol to form phosphatidate. This enzyme, in conjunction with *PAH1*-encoded phosphatidate phosphatase, controls the levels of phosphatidate and diacylglycerol for phospholipid synthesis, membrane growth, and lipid droplet formation. In this work, we showed that a functional level of diacylglycerol kinase is regulated by the Reb1p transcription factor. In the electrophoretic mobility shift assay, purified recombinant Reb1p was shown to specifically bind its consensus recognition sequence (CGGGTAA, −166 to −160) in the *DGK1* promoter. Analysis of cells expressing the P*_DGK1_-lacZ* reporter gene showed that mutations (GT→TG) in the Reb1p-binding sequence caused an 8.6-fold reduction in β-galactosidase activity. The expression of *DGK1*(reb1), a *DGK1* allele containing the Reb1p-binding site mutation, was greatly lower than that of the wild type allele, as indicated by analyses of *DGK1* mRNA, Dgk1p, and diacylglycerol kinase activity. In the presence of cerulenin, an inhibitor of *de novo* fatty acid synthesis, the *dgk1*Δ mutant expressing *DGK1*(reb1) exhibited a significant defect in growth as well as in the synthesis of phospholipids from triacylglycerol mobilization. Unlike *DGK1*, the *DGK1*(reb1) expressed in the *dgk1*Δ *pah1*Δ mutant did not result in the nuclear/endoplasmic reticulum membrane expansion, which occurs in cells lacking phosphatidate phosphatase activity. Taken together, these results indicate that the Reb1p-mediated regulation of diacylglycerol kinase plays a major role in its *in vivo* functions in lipid metabolism.

## Teaser

**Background:** Diacylglycerol kinase produces phosphatidate, a major precursor for the synthesis of membrane phospholipids.

**Results:** The expression of diacylglycerol kinase is induced by the Reb1p transcription factor, and the resulting activity increase is essential for the enzyme function in phospholipid synthesis.

**Conclusion:** The Reb1p-mediated transcriptional activation regulates the expression of diacylglycerol kinase activity.

**Significance:** Diacylglycerol kinase is regulated at the level of transcription.

## Introduction

**FIGURE 1.**
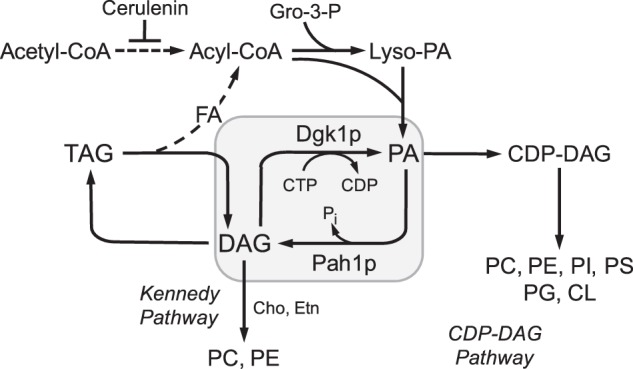
**Mobilization of TAG for phospholipid synthesis.** The figure shows an abbreviated pathway for the mobilization of TAG for phospholipid synthesis when fatty acid synthesis is blocked with cerulenin. The metabolism of PA and DAG, as catalyzed by Dgk1p DAG kinase and Pah1p PA phosphatase, is highlighted by *gray shading. FA*, fatty acid; *Gro-3-P*, glycerol 3-phosphate; *PC*, phosphatidylcholine; *PE*, phosphatidylethanolamine; *PI*, phosphatidylinositol; *PS*, phosphatidylserine; *PG*, phosphatidylglycerol; *CL*, cardiolipin; *Cho*, choline; *Etn*, ethanolamine.

In the budding yeast *Saccharomyces cerevisiae*, the *DGK1*-encoded DAG^2^ kinase is an ER-associated enzyme that catalyzes the formation of PA from DAG (1, 2). In contrast to the ATP-dependent DAG kinase enzymes from animals, plants, and bacteria (3–7), the yeast enzyme uses CTP instead of ATP as the phosphate donor in the reaction (Fig. 1) (1, 2). The DAG kinase is an important enzyme because its substrate and product are intermediates in the synthesis and turnover of membrane phospholipids and the lipid droplet constituent TAG (8–11). In addition, PA and DAG are signaling molecules that influence transcription, membrane proliferation, vesicular trafficking, and cell growth (5, 12–20). The importance of DAG kinase in mammalian cell physiology is emphasized by the fact that the α isoform has been identified as a therapeutic target in glioblastoma and other cancers (21).

Maintenance of the ER membrane PA/DAG balance is critical to the physiology of *S. cerevisiae*, and DAG kinase and *PAH1*-encoded PA phosphatase (enzyme that catalyzes the conversion of PA to DAG (Fig. 1) (22)) play essential roles in this process (1, 23). For example, a disturbance in the PA/DAG balance, as controlled by these two enzymes, results in the abnormal regulation of phospholipid synthesis gene expression and phospholipid content, the aberrant growth of the nuclear/ER membrane, vacuole fragmentation, and a defect in lipid droplet formation (1, 22, 24–33). In addition, DAG kinase facilitates cellular health by alleviating the toxic effects caused by DAG (34, 35).

DAG kinase also plays an important role in the metabolic process whereby yeast cells in stasis (*i.e.* stationary phase) resume vegetative growth upon nutrient supplementation (34). This process, especially when *de novo* fatty acid synthesis is inhibited, is dependent on the hydrolysis of TAG (mediated by Tgl3p and Tgl4p TAG lipases) to generate fatty acids for initiation of membrane phospholipid synthesis (Fig. 1) (10, 11, 36–39). The DAG kinase utilizes the TAG-derived DAG to generate PA for phospholipid synthesis via the liponucleotide intermediate CDP-DAG (Fig. 1) (34). The CDP-DAG-dependent pathway is the principal route by which all major membrane phospholipids are synthesized in *S. cerevisiae* (8, 9). The role for DAG kinase can be partially substituted by channeling DAG into phosphatidylcholine and phosphatidylethanolamine synthesized via the Kennedy pathway (8, 9) by supplementation of choline or ethanolamine to the growth medium (Fig. 1) (34).

In this work, we showed that the expression of *DGK1* is regulated by the transcription factor Reb1p (RNA polymerase I enhancer-binding protein). Mutations in the Reb1p-binding site blocked the interaction of Reb1p with the *DGK1* promoter resulting in a decrease in *DGK1* expression. Loss of Reb1p-mediated regulation of DAG kinase compromised the PA/DAG balance, as reflected in nuclear/ER membrane growth, and the metabolic process of TAG mobilization for membrane phospholipid synthesis and the resumption of growth from stasis. This work advanced the understanding of the regulation of *DGK1* as well as the role Reb1p plays in the regulation of lipid metabolism.

## EXPERIMENTAL PROCEDURES

### 

#### 

##### Materials

All chemicals were reagent grade. Difco was the source of growth medium components. Restriction endonucleases, modifying enzymes, and Phusion high fidelity DNA polymerase were from New England Biolabs. Qiagen was the supplier of the DNA purification kit and nickel-nitrilotriacetic acid-agarose resin. Clontech was the source of the yeast transformation kit. Genosys Biotechnology, Inc., was the supplier of oligonucleotides used for PCRs and electrophoretic mobility shift assays. Cerulenin, nucleotides, IGEPAL CA-630, nucleoside 5′-diphosphate kinase, Triton X-100, and protease inhibitors (phenylmethylsulfonyl fluoride, benzamidine, aprotinin, leupeptin, and pepstatin) were from Sigma. PerkinElmer Life Sciences and National Diagnostics were the sources of radiochemicals and scintillation counting supplies, respectively. Lipids were obtained from Avanti Polar Lipids, and silica gel TLC plates were from EM Science. Protein assay reagents, electrophoresis reagents, DNA and protein size standards, and iScript One-Step RT-PCR kit with SYBR Green were from Bio-Rad. Invitrogen was the source of the Ambion TURBO DNA-free kit. ProbeQuant G-50 micro columns, polyvinylidene difluoride membrane, and the enhanced chemifluorescence Western blot reagent were purchased from GE Healthcare. Roche Applied Science supplied the mouse anti-HA and anti-His_6_ antibodies.

##### Strains and Growth Conditions

The strains used in this work are listed in Table 1. Yeast cells were grown in YEPD medium (1% yeast extract, 2% peptone, 2% glucose) or in SC medium containing 2% glucose at 30 °C as described previously (40, 41). For selection of yeast cells bearing plasmids, the appropriate amino acids were omitted from SC medium. Plasmid maintenance/amplifications (strain DH5α) and Reb1p expression (strain BL21(DE3)pLysS) were performed in *Escherichia coli*. The bacterial cells were grown in LB medium (1% tryptone, 0.5% yeast extract, 1% NaCl (pH 7.4)) at 37 °C, and ampicillin (100 μg/ml) was added to select for the cells carrying plasmid. For growth on solid media, agar plates were prepared with supplementation of either 2% (yeast) or 1.5% (*E. coli*) agar. For heterologous expression of His_6_-tagged Reb1p, *E. coli* BL21(DE3)pLysS cells bearing pYQ3 were grown to *A*_600 nm_ = 0.5 at 30 °C in 1 liter of LB medium containing ampicillin (100 μg/ml) and chloramphenicol (34 μg/ml). The culture was then incubated for 3 h with 0.5 mm isopropyl β-d-thiogalactoside to induce the expression of Reb1p.

**TABLE 1:** **Stains and plasmids used in this study**

Strain or plasmid	Relevant characteristics	Source or Ref.
**Strain**		
*E. coli*		
DH5α	F^−^ φ80d*lacZ*ΔM15Δ (*lacZYA-argF*)U169 *deoR recA1 endA1 hsdR17* (*r_k_*^−^ *m_k_*^+^) *phoA supE44* l^−^*thi-1 gyrA96 relA1*	41
BL21(DE3)pLysS	F^−^ *ompT hsdS_B_* (*r_B_*^−^*m_B_*^−^) *gal dcm* (DE3) pLysS	Novagen
*S. cerevisiae*		
RS453	*MAT***a** *ade2-1 his3-11,15 leu2-3,112 trp1-1 ura3-52*	100
SS1144	*dgk1*Δ::*HIS3* derivative of RS453	1
SS1147	*dgk1*Δ::*HIS3 pah1*Δ::*TRP1* derivative of RS453	1

**Plasmid**		
pRS416	Low copy *E. coli*/yeast shuttle vector with *URA3*	101
pSF211	*DGK1* inserted into pRS416	34
pSF213	Derivative of pSF211 with GT→TG mutations in the Reb1p-binding site	This study
pJO2	P*_DPP1_-lacZ* reporter gene with *URA3*	46
pYQ1	P*_DGK1_-lacZ* reporter gene with *URA3*	This study
pYQ2	Derivative of pYQ1 with GT→TG mutations in the Reb1p-binding site	This study
pET-15b	*E. coli* expression vector with the N-terminal His_6_ tag fusion	Novagen
pYQ3	*REB1* coding sequence inserted into pET-15b	This study
YCplac33-SEC63-GFP	*SEC63*-GFP fusion into the *CEN/URA3* vector	1
YCplac33-PAH1	*PAH1* into the *CEN/URA3* vector	24

The growth regime of Fakas *et al.* (34) was used to examine the effects of the Reb1p-binding site mutation on the resumption of growth from the stationary phase. Cultures were grown for 48 h in SC medium to reach stationary phase, harvested by centrifugation, and diluted with fresh SC medium. Cerulenin (10 μg/ml) was added to the cultures to inhibit fatty acid synthesis (42, 43). For growth curves, cultures (200 μl) were incubated in 96-well plates, and the cell density was monitored at *A*_650 nm_ with a Thermomax plate reader. Generation times were calculated from the growth curves according to the modified Gompertz equation (44).

##### DNA Manipulations, Amplification of DNA by PCR, Construction of Plasmids, and DNA Sequencing

Standard methods were used to isolate plasmid and genomic DNA and for the manipulation of DNA using restriction enzymes, DNA ligase, and modifying enzymes (41). PCRs were optimized as described by Innis and Gelfand (45). The plasmids used in this work are listed in Table 1. Plasmid pSF213, which was derived from plasmid pSF211, contains *DGK1* with two transversion mutations in the Reb1p-binding site. This plasmid was constructed by PCR-mediated site-directed mutagenesis (primers: forward, 5′-ATCCAGGGTCCATAGCGGTGAACAAATTATTGGTT-3′; reverse, 5′-AACCAATAATTTGTTCACCGCTATGGACCCTGGAT-3′). Plasmid pSF211 was eliminated from the reaction by digestion with DpnI. Plasmids pYQ1 and pYQ2 contain the wild type and mutant *DGK1* promoters, respectively, fused to the coding sequence of the *lacZ* gene of *E. coli*. They were constructed by replacing the *DPP1* promoter in pJO2 (46) with the wild type and mutant *DGK1* promoter sequences at the EcoRI site. These *DGK1* promoter sequences were obtained by PCR (primers: forward, 5′-GAGCTCGAATTCTCGTTTACCAACTGAA-3′; reverse 5′-GAGCTCGAATTCATATTGTCTGTAAACCC-3′) using plasmids pSF211 and pSF213, respectively, as the templates. For expression of Reb1p in *E. coli*, the *REB1* coding sequence was amplified by PCR (primers: forward, 5′-CAGCCATATGCCTTCAGGTCATAACGATAAA-3′; reverse, 5′-GCCGGATCCTCGAGTTAATTTTCTGTTTTCATTGA-3′) using strain RS453 genomic DNA as the template. The 2,448-bp PCR product was digested with NdeI and XhoI, and the product was ligated into pET-15b at its NdeI/XhoI sites. The resulting plasmid that bears the His_6_-tagged *REB1* was named pYQ3. All plasmid constructions were verified by DNA sequencing, which was performed by GENEWIZ, Inc. Standard protocols were used to transform *E. coli* (41) and yeast (47) cells with plasmids.

##### RNA Isolation and Quantitative RT-PCR

Total RNA was isolated with hot phenol (48–50) and treated with the Ambion TURBO DNA-free kit to remove DNA contamination. *DGK1* cDNA was synthesized and amplified on a Bio-Rad MyiQ single-color real time PCR detection system using the iScript one-step RT-PCR kit with SYBR Green and *DGK1* primers (forward, 5′-CACCCAAAGTGGCAAGAAAT-3′; reverse, 5′-AAGCAGCTACCACACCACCT-3′). Quantification of each measurement was determined from a standard curve generated by PCR amplification run simultaneously with the RT-PCRs from plasmid pSF211 of known copy number. Each sample was run in triplicate, and the PCR efficiency was 80–90%. Reactions without reverse transcriptase were included as a control for DNA contamination.

##### Electrophoretic Mobility Shift Assays

Double-stranded oligonucleotides for the wild type (5′-AGGGTCCATAGCGGGTAACAAATTATTGG-3′/3′-TCCCAGGTATCGCCCATTGTTTAATAACC-5′) and mutant (5′-AGGGTCCATAGCGGTGAACAAATTATTGG-3′/3′-TCCCAGGTATCGCCACTTGTTTAATAACC-5′) sequences for Reb1p binding were prepared, labeled with [α-^32^P]dTTP (400–800 Ci/mmol) and Klenow fragment (5 units), and then purified by gel filtration using ProbeQuant G-50 spin columns as described previously (51). The radiolabeled DNA probe (4 pmol, 8.0 × 10^4^ cpm/pmol) and purified recombinant His_6_-Reb1p were mixed in a total reaction volume of 10 μl and were incubated for 15 min at room temperature. The reaction buffer contained 10 mm Tris-HCl (pH 8.0), 10 mm MgCl_2_, 50 mm KCl, 1 mm dithiothreitol, 0.025 mg/ml poly(dI-dC)·poly(dI-dC), 0.2 mg/ml bovine serum albumin, 0.04% IGEPAL CA-630, and 10% glycerol. Following incubation, the reaction mixture was resolved for 45 min at 100 V on a 5% polyacrylamide gel (1.5-mm thickness) in 0.5× Tris borate/EDTA buffer. Gels were dried onto a filter paper, and the radioactive signals were visualized by phosphorimaging analysis.

##### Purification of His_6_-tagged Reb1p

**FIGURE 2.**
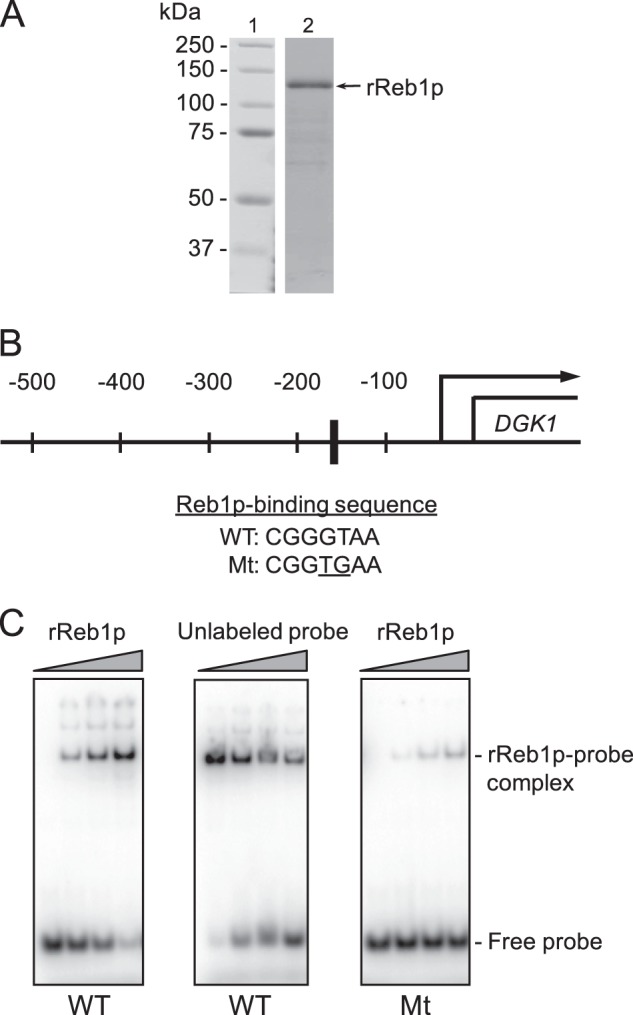
**Interactions of Reb1p with its putative binding site in the *DGK1* promoter.** *A*, purified preparation (2 μg) of the His_6_-tagged recombinant Reb1p (*rReb1p*) was subjected to SDS-PAGE and stained with Coomassie Blue. The positions of the molecular mass standards (*lane 1*) and the purified rReb1p (*lane 2*) are indicated. *B,* location (−166 to −160) and sequence of the putative Reb1p-binding site in the *DGK1* promoter. Also shown is the sequence of the mutant (*Mt*) form of the Reb1p-binding site. *C,* recombinant His_6_-Reb1p was mixed with 4 pmol of radiolabeled double-stranded oligonucleotide (8.0 × 10^4^ cpm/pmol) with or without mutations in the Reb1p-binding site. The *left* and *right panels* show assays with 0, 0.2, 0.5, and 1.0 μg of recombinant His_6_-Reb1p. The *middle panel* shows an assay with 0.5 μg of recombinant His_6_-Reb1p in the presence of 0, 4, 8, and 16 pmol of unlabeled wild type oligonucleotide. Mixtures of His_6_-Reb1p with oligonucleotide probes were subjected to electrophoresis in a 5% polyacrylamide gel. The data shown are representative of two independent experiments. The positions of the Reb1p-Reb1p-binding site complex and free oligonucleotide probe are indicated in the figure.

His_6_-tagged Reb1p expressed in *E. coli* BL21(DE3)pLysS was purified to near homogeneity by affinity chromatography using nickel-nitrilotriacetic acid-agarose (52). As described previously (53), the recombinant Reb1p with the predicted molecular mass of 92 kDa migrated on an 8% SDS-polyacrylamide gel as a 127-kDa protein (Fig. 2). Purified His_6_-Reb1p was stored at −80 °C.

##### Preparation of Cell Extracts and Total Membranes and Protein Determination

All steps were performed at 4 °C. Cell extracts were prepared by disruption of yeast cells with glass beads (0.5 mm diameter) using a BioSpec Products Mini-BeadBeater-16 (54). The cell disruption buffer contained 50 mm Tris-HCl (pH 7.5), 0.3 m sucrose, 10 mm 2-mercaptoethanol, 0.5 mm phenylmethanesulfonyl fluoride, 1 mm benzamidine, 5 μg/ml aprotinin, 5 μg/ml leupeptin, and 5 μg/ml pepstatin. The total membrane fraction (pellet) was prepared by centrifugation of the cell extract at 100,000 × *g* for 1 h (54). Protein concentration was estimated by the Coomassie Blue dye-binding method of Bradford (55) using bovine serum albumin as the standard.

##### SDS-PAGE and Western Blot Analysis

SDS-PAGE (56) using 12% slab gels and Western blotting (57, 58) using polyvinylidene difluoride membrane were performed as described previously. Proteins in polyacrylamide gels were visualized by staining with Coomassie Blue R-250. The polyvinylidene difluoride membrane blots were probed with anti-Dgk1p antibodies (2) or with anti-Dpp1p antiserum (59) at a concentration of 1 μg/ml and a dilution of 1:1000, respectively, followed by goat anti-rabbit IgG antibodies conjugated with alkaline phosphatase (dilution of 1:5,000). The immune complexes were detected using the enhanced chemifluorescence Western blotting detection kit. Fluorimaging was used to acquire fluorescent signals from the immune complex reactions.

##### Enzyme Assays

DAG kinase activity was measured by following the incorporation of the γ-phosphate of water-soluble [γ-^32^P]CTP (70,000 cpm/nmol) into chloroform-soluble PA as described previously (2). The reaction mixture contained 50 mm Tris-HCl (pH 7.5), 0.1 mm dioleoyl-DAG, 1 mm Triton X-100, 1 mm CTP, 1 mm CaCl_2_, 10 mm 2-mercaptoethanol, and enzyme protein in a total volume of 0.1 ml. The [γ-^32^P]CTP used in the reaction was synthesized enzymatically from CDP and [γ-^32^P]ATP with nucleoside 5′-diphosphate kinase (60). β-Galactosidase activity was measured by following the formation of *O-*nitrophenyl from *O-*nitrophenyl β-d-galactopyranoside at *A*_410 nm_ (61). The reaction mixture contained 100 mm sodium phosphate (pH 7.0), 3 mm *O-*nitrophenyl β-d-galactopyranoside, 1 mm MgCl_2_, 100 mm 2-mercaptoethanol, and enzyme protein in a total volume of 0.1 ml. All enzyme assays were conducted in triplicate at 30 °C. The enzyme assays were linear with time and protein concentration. The units of DAG kinase and β-galactosidase activities were defined as the amount of enzymes that catalyzed the formation of 1 pmol of product/min and 1 nmol of product/min, respectively.

##### Labeling and Analysis of Lipids

Cells were grown to stationary phase in the presence of [2-^14^C]acetate (1 μCi/ml) to uniformly label lipids. The labeled stationary phase cells were washed with water and resuspended to an *A*_600 nm_ of 0.5 in fresh growth medium without label to follow the mobilization of TAG (34). Total lipids were extracted (62) and analyzed by TLC using the solvent system hexane/diethyl ether/glacial acetic acid (40:10:1, v/v/v). The identity of labeled lipids on TLC plates was confirmed by comparison with standards after exposure to iodine vapor. Radiolabeled lipids were visualized by phosphorimaging analysis, and the relative quantities of labeled lipids were analyzed using ImageQuant software.

##### Microscopy

Cells grown at 30 °C in SC medium lacking leucine and uracil were collected at mid-exponential phase, resuspended in a reduced volume of the same medium, and immediately imaged live at room temperature. Images were acquired with an epifluorescence microscope (Zeiss Axioplan) using a 100× plan-apochromatic 1.4NA objective lens (Carl Zeiss Ltd.), connected to a Hamamatsu Orca R2 CCD camera and controlled by the Simple PCI6 software (Hamamatsu). The brightness and contrast of the resulting images were adjusted using Adobe Photoshop.

##### Data Analyses

Student's *t* test (SigmaPlot software) was used to determine statistical significance, and *p* values of <0.05 were taken as a significant difference.

## RESULTS

### 

#### 

##### Reb1p Interacts with a Reb1p-binding Site in the DGK1 Promoter

The *DGK1* promoter contains the core consensus sequence (CGGGTAA, −166 to −160) for binding of the transcription factor Reb1p (53, 63–65). To determine whether the *DGK1* sequence interacts with Reb1p, we performed an electrophoretic mobility shift assay with a double-stranded oligonucleotide probe containing the recognition sequence and pure His_6_-tagged Reb1p (Fig. 2*A*). The radiolabeled probe showed a decreased electrophoretic mobility in a dose-dependent manner with respect to Reb1p (Fig. 2*C*, *left panel*). Unlabeled probe competed with the labeled probe for Reb1p binding in a dose-dependent manner (Fig. 2*C*, *middle panel*), indicating the specificity of the protein-DNA interaction. However, when transverse mutations (GT→TG, Fig. 2*B*) that are known to abolish Reb1p binding to the Reb1p-binding sequence (66) were introduced into the binding site, the electrophoretic mobility shift of the probe was greatly attenuated (Fig. 2*C*, *right panel*). Taken together, these data supported the conclusion that Reb1p directly interacts with the Reb1p-binding sequence in the *DGK1* promoter.

##### Reb1p-binding Site Mutation Attenuates the Expression of P_DGK1_-lacZ Reporter Gene Activity and the Abundance of DGK1 mRNA

**FIGURE 3.**
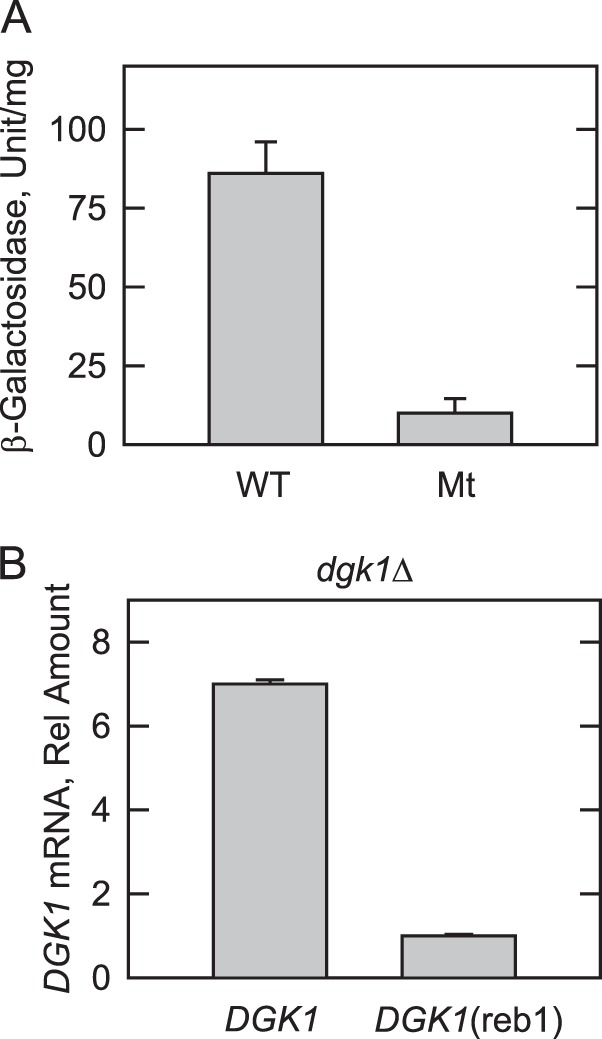
**Effect of the Reb1p-binding site mutation on P*_DGK1_-lacZ* expression and *DGK1* mRNA abundance.** *A*, wild type cells bearing the wild type P*_DGK1_-lacZ* or the mutant (*Mt*) P*_DGK1_*_(reb1)_-*lacZ* reporter gene were grown in SC medium to the exponential phase; cell extracts were prepared and assayed for β-galactosidase activity. Each data point represents the average of triplicate enzyme determinations from five independent experiments ± S.D. (*error bars*). *B*, *dgk1*Δ cells expressing *DGK1* or *DGK1*(reb1) from low copy plasmids were grown in SC medium to the exponential phase. Total RNA was isolated, and the absolute levels of *DGK1* mRNA were quantified by real time reverse transcription-PCR. The level of *DGK1* mRNA from the *DGK1* expression is expressed relative to that from the *DGK1*(reb1) expression. Each data point represents the average of triplicate determinations from two independent experiments ± S.D. (*error bars*).

The expression of *DGK1* was examined by use of a P*_DGK1_-lacZ* reporter gene where the *DGK1* promoter was fused with the coding sequence of the *lacZ* gene of *E. coli*. The β-galactosidase activity was dependent on the transcription of *lacZ* driven by the *DGK1* promoter. The β-galactosidase activity in wild type exponential phase cells expressing the reporter gene was 86 ± 11 nmol/min/mg. The Reb1p-binding site mutation in the P*_DGK1_-lacZ* reporter gene reduced the β-galactosidase activity by 8.6-fold (Fig. 3*A*). By using quantitative RT-PCR, we also examined whether Reb1p controls *DGK1* transcription in exponential phase cells. The amount of *DGK1* mRNA of *DGK1*(reb1)-expressing *dgk1*Δ cells was 7-fold lower when compared with that of cells expressing the wild type *DGK1* allele (Fig. 3*B*).

##### Reb1p-binding Site Mutation Abolishes the DGK1-mediated Nuclear/ER Membrane Expansion

**FIGURE 4.**
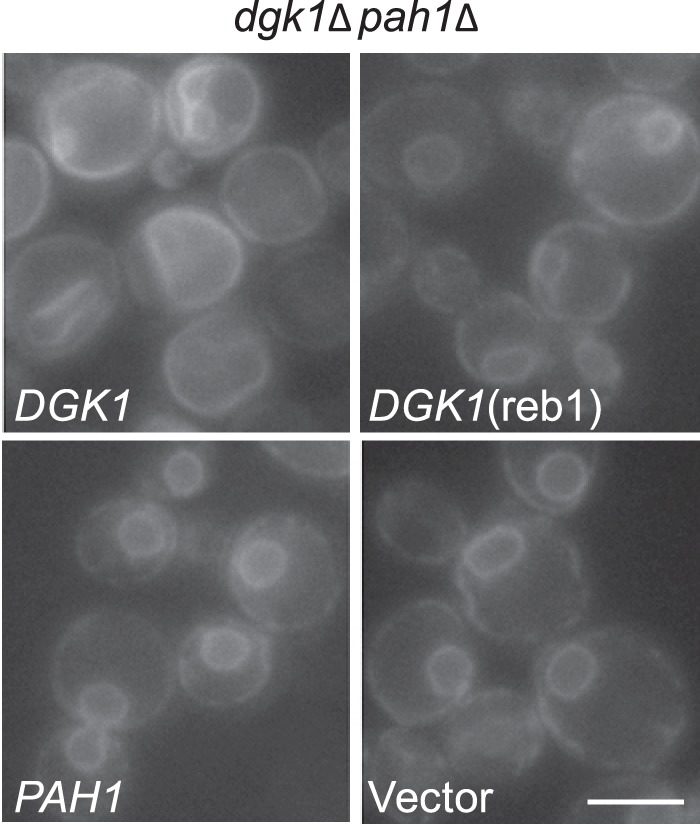
**Effect of the Reb1p-binding site mutation on the nuclear/ER membrane structure of cells lacking *DGK1* and *PAH1*.** *dgk1*Δ *pah1*Δ cells expressing *SEC63-GFP* (to label the nuclear/ER membrane) and *DGK1*, *DGK1*(reb1), or *PAH1* from low copy plasmids were grown in SC medium to the exponential phase of growth. The fluorescence signal from the reporter protein was examined with a Zeiss Axioplan epifluorescence microscope equipped with a 100× plan apochromatic 1.4NA objective lens. The *white bar* indicates 5 μm.

Expression of the *DGK1* gene is required for the aberrant expansion of the nuclear/ER membrane when the *PAH1* gene is deleted (1). The basis for this phenotype is that the *DGK1*-encoded DAG kinase activity causes the accumulation of PA at the nuclear/ER membrane when the phospholipid is not hydrolyzed by *PAH1*-encoded PA phosphatase (1). To examine the dependence of *DGK1* expression and function of the Reb1p-binding site *in vivo*, we examined the effect of the Reb1p-binding site mutation on nuclear/ER membrane expansion. For this experiment, the *DGK1* and *DGK1*(reb1) alleles were expressed in *dgk1*Δ *pah1*Δ cells. Expression of *DGK1* in the double mutant caused membrane expansion, whereas the Reb1p-binding site mutation did not (Fig. 4). As described previously (1), the *dgk1*Δ mutation alone (*i.e. dgk1*Δ *pah1*Δ/*PAH1*) and in combination with the *pah1*Δ mutation (*i.e. dgk1*Δ *pah1*Δ/vector) did not cause the aberrant nuclear/ER membrane expansion (Fig. 4). This result indicated that the Reb1p-mediated regulation of *DGK1* expression is crucial for its cellular function.

##### Reb1p-binding Site Mutation Compromises Growth Resumption from Stationary Phase in the Presence of Cerulenin

**FIGURE 5.**
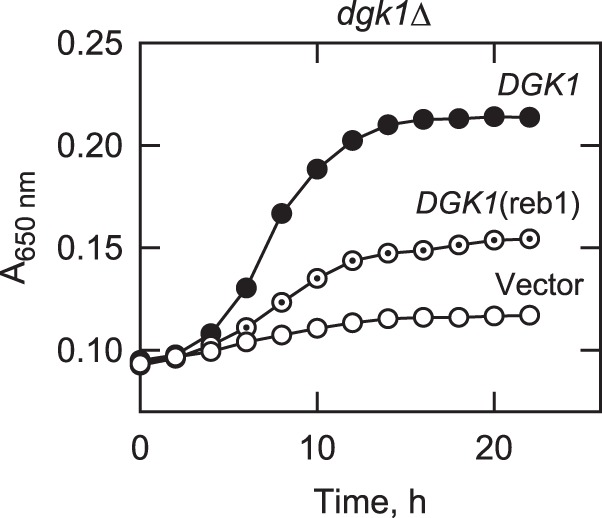
**Effect of the Reb1p-binding site mutation on the resumption of cell growth from stationary phase in the absence of *de novo* fatty acid synthesis.** *dgk1*Δ cells expressing the *DGK1* and *DGK1*(reb1) from low copy plasmids were grown to stationary phase in SC medium and then diluted in fresh medium containing 10 μg/ml cerulenin. Cell growth after the transfer to fresh medium was monitored with a plate reader. Each data point represents the average of three independent cultures. The generation times for *dgk1*Δ cells expressing *DGK1*, *DGK1*(reb1), and vector were 17.4 ± 0.4, 50.2 ± 1.4, and 133.2 ± 0.7 h, respectively.

Stationary phase (static) cells resume vegetative growth upon replenishment with nutrients, and this process is dependent on the mobilization of TAG to synthesize phospholipids (34, 36, 67). Resumption of growth following stasis is dependent on *DGK1* when fatty acid synthesis is blocked because the conversion of TAG-derived DAG to PA is needed for phospholipid synthesis (34). Accordingly, we questioned if Reb1p-mediated *DGK1* expression was important for growth resumption from stationary phase. *dgk1*Δ cells expressing the wild type *DGK1* and *DGK1*(reb1) alleles were first grown to the stationary phase and then allowed to grow in fresh medium containing cerulenin, an inhibitor for fatty acid synthesis (42). As described previously (34), the expression of the wild type *DGK1* gene complemented the loss-of-growth phenotype exhibited by *dgk1*Δ mutant cells (Fig. 5). However, the expression of the *DGK1*(reb1) mutant allele only partially complemented the growth defect (Fig. 5). The generation time (50.2 ± 1.4 h) of cells expressing *DGK1*(reb1) was 2.9 times longer than the generation time (17.4 ± 0.4 h) of cells expressing the wild type *DGK1* gene. Thus, the Reb1p-mediated expression of *DGK1* was important for growth resumption from stasis.

##### Reb1p-binding Site Mutation Attenuates Expression of P_DGK1_-lacZ Reporter Gene Activity, Dgk1p, and DAG Kinase Activity upon Nutrient Supplementation of Stationary Phase Cells

**FIGURE 6.**
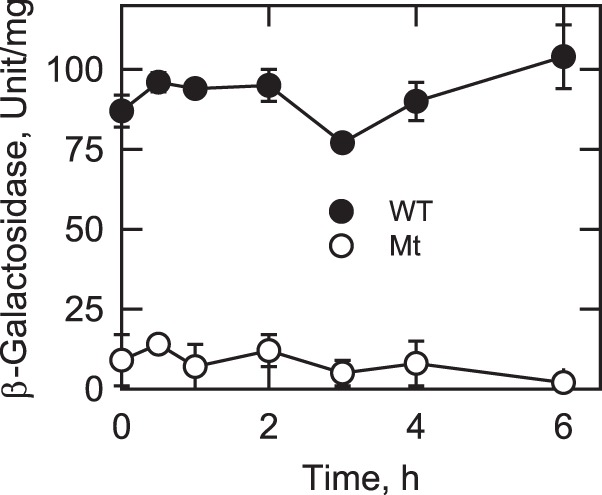
**Effect of the Reb1p-binding site mutation on P*_DGK1_-lacZ* expression upon growth resumption from stationary phase in the absence of *de novo* fatty acid synthesis.** Wild type cells bearing the wild type P*_DGK1_-lacZ* or the mutant (*Mt*) P*_DGK1_*_(reb1)_-*lacZ* reporter gene were grown to stationary phase in SC medium and then diluted in fresh medium containing 10 μg/ml cerulenin. At the indicated time intervals, cells were harvested; cell extracts were prepared and assayed for β-galactosidase activity. Each data point represents the average of triplicate enzyme determinations from five independent experiments ± S.D. (*error bars*).

To provide mechanistic information for the attenuation of growth in cells expressing the *DGK1*(reb1) allele, the *DGK1* promoter activity was measured during growth resumption from stasis. For these experiments, *DGK1* promoter activity was monitored by the β-galactosidase activity from the P*_DGK1_-lacZ* reporter gene expression. At stationary phase (at 0 h), the β-galactosidase activity of cells expressing P*_DGK1_*_(reb1)_-*lacZ* was 9.6-fold lower than the activity of cells expressing the wild type reporter gene (Fig. 6). Although there was a relatively small variation in the β-galactosidase activity of cells expressing the wild type reporter gene after nutrient supplementation, the level of expression was fairly constant during the course of the experiment. Likewise, the much reduced level of β-galactosidase activity from the mutant reporter gene expression was moderately constant after nutrient supplementation (Fig. 6).

**FIGURE 7.**
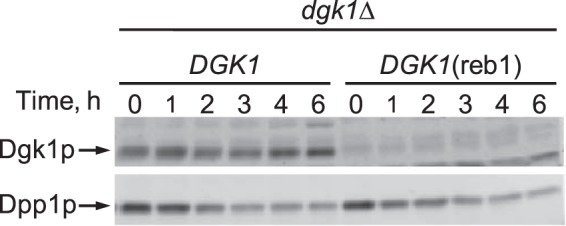
**Effect of the Reb1p-binding site mutation on Dgk1p abundance upon growth resumption from stationary phase in the absence of *de novo* fatty acid synthesis.** *dgk1*Δ cells expressing *DGK1* and *DGK1*(reb1) from low copy plasmids were grown to stationary phase in SC medium and then diluted in fresh medium containing 10 μg/ml cerulenin. At the indicated time intervals, cells were harvested; total membranes (20 μg) were prepared, and the amount of Dgk1p was determined by Western blot analysis using anti-Dgk1p antibodies. The same blot was also probed with anti-Dpp1p antibodies to detect the *DPP1*-encoded DAG pyrophosphate phosphatase. Portions of representative blots from three experiments are shown in the figure, and the positions of Dgk1p and Dpp1p are indicated.

Next, we questioned whether the Reb1p-mediated control of *DGK1* expression is translated into the levels of Dgk1p. Western blot analysis showed that there was some variation in the level of the Dgk1p at different time points (Fig. 7). However, the major conclusion from this experiment was that the levels of Dgk1p in cells expressing the *DGK1*(reb1) allele were greatly reduced (∼7-fold) when compared with cells expressing the wild type *DGK1* gene (Fig. 7). To confirm that the levels of Dgk1p were from cells at different growth phases, we analyzed the levels of *DPP1*-encoded DAG pyrophosphate phosphatase (Dpp1p) whose expression is known to be elevated in stationary phase and reduced in exponential phase (59). The growth phase-mediated regulation of Dpp1p expression was not altered in the *dgk1*Δ cells expressing *DGK1* and *DGK1*(reb1) (Fig. 7).

**FIGURE 8.**
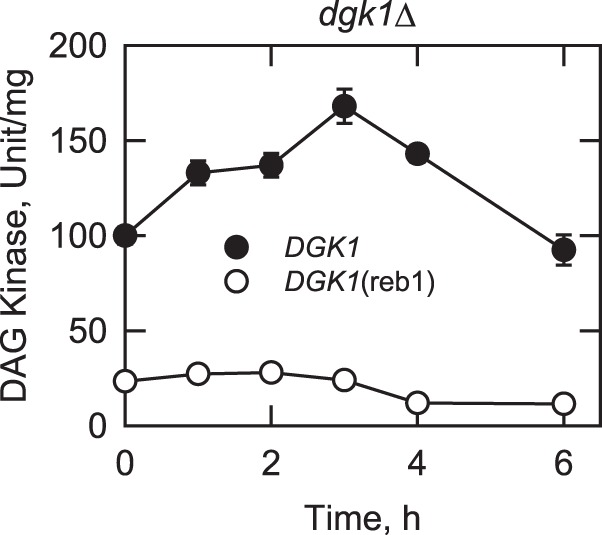
**Effect of the Reb1p-binding site mutation on DAG kinase activity upon growth resumption from stationary phase in the absence of *de novo* fatty acid synthesis.** *dgk1*Δ cells expressing *DGK1* and *DGK1*(reb1) from low copy plasmids were grown to stationary phase in SC medium and then diluted in fresh medium containing 10 μg/ml cerulenin. At the indicated time intervals, cells were harvested; cell extracts were prepared, and DAG kinase activity was measured. Each data point represents the average of triplicate enzyme determinations from a minimum of two independent experiments ± S.D. (*error bars*).

The effect of the Reb1p-binding site mutation on the *DGK1* expression was also examined by analysis of DAG kinase activity (Fig. 8). In stationary phase cells (at 0 h), the enzyme activity in *dgk1*Δ cells expressing the *DGK1*(reb1) allele was 4.3-fold lower than cell expressing *DGK1*. The reduction in the levels of DAG kinase activity correlated with the reduction in the expression levels of the reporter gene and Dgk1p. As described previously (34), a transient increase (∼1.7-fold) was shown in the level of DAG kinase activity when *dgk1*Δ cells expressing *DGK1* resumed vegetative growth from stasis (Fig. 8). However, the reduced level of DAG kinase activity in cells expressing the *DGK1*(reb1) allele did not show change (Fig. 8).

##### Reb1p-binding Site Mutation Compromises the Mobilization of TAG for Phospholipid Synthesis upon Nutrient Supplementation of Stationary Phase Cells

**FIGURE 9.**
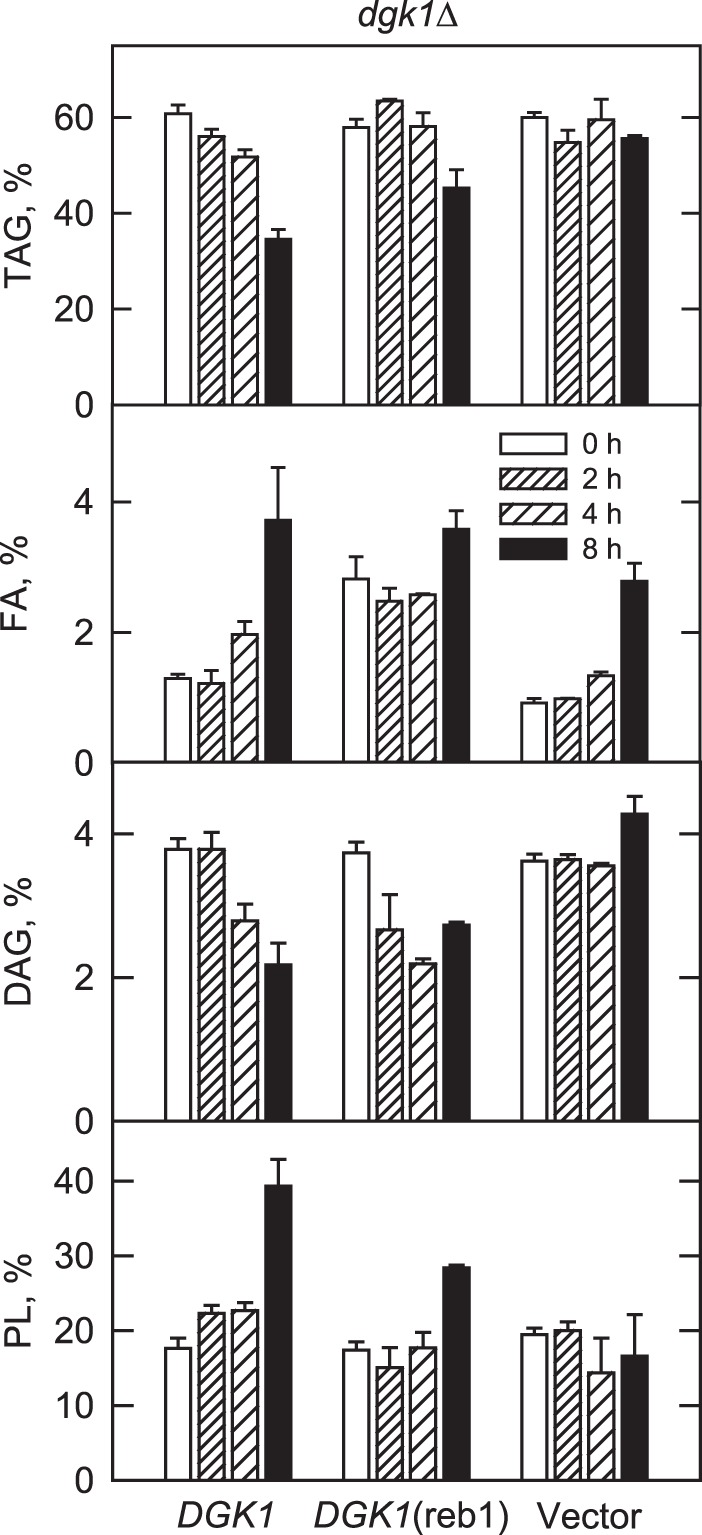
**Effect of the Reb1p-binding site mutation on the mobilization of TAG for phospholipid synthesis upon growth resumption from stationary phase in the absence of *de novo* fatty acid synthesis.** *dgk1*Δ cells expressing the *DGK1* and *DGK1*(reb1) from low copy plasmids were grown to stationary phase in SC medium in the presence of [2-^14^C]acetate (1 μCi/ml) to uniformly label cellular lipids. The cells were then washed to remove the label and resuspended in fresh medium containing 10 μg/ml cerulenin. At the indicated time intervals, cells were harvested, and lipids were extracted and separated by one-dimensional TLC. The ^14^C-labeled lipids were visualized by phosphorimaging and quantified by ImageQuant analysis. The percentages shown for the individual lipids were normalized to the total ^14^C-labeled chloroform-soluble fraction. The values reported are the average of five separate experiments ± S.D. (*error bars*). *FA*, fatty acids; *PL*, phospholipids.

To examine the role of the Reb1p-mediated expression of *DGK1* in the resumption of growth from stationary phase when fatty acid synthesis is blocked, the mobilization of TAG to phospholipids was followed by a [2-^14^C]acetate labeling chase experiment (34). The amounts of lipids was determined up to 8 h following nutrient supplementation because maximum TAG hydrolysis has been shown in this time frame (34). As described previously (34), the mobilization of TAG was not shown in *dgk1*Δ mutant cells. This metabolic defect, however, was complemented by expression of the wild type *DGK1* allele (Fig. 9). In *dgk1*Δ cells expressing *DGK1*, the amount of TAG declined in a time-dependent manner to a maximum of 43% by 8 h (Fig. 9). Reciprocally, the amount of phospholipids increased in a time-dependent manner to a maximum of 100% by 8 h (Fig. 9). Over this time period, the level of fatty acids increased by 186%, whereas the level of DAG decreased by 70% (Fig. 9). However, the Reb1p-binding site mutation attenuated the mobilization of TAG; the reduction in TAG content was only 22% and the increase in phospholipids was 78% by 8 h after the nutrient supplementation (Fig. 9).

## DISCUSSION

The *DGK1*-encoded CTP-dependent DAG kinase has emerged as an important lipid metabolic enzyme in *S. cerevisiae* (1, 2, 30, 34). This ER-associated enzyme plays an important role in controlling the PA/DAG balance in the nuclear/ER membrane, which in turn regulates the synthesis of phospholipids, membrane growth, and lipid droplet formation (1, 2, 30). DAG kinase also alleviates the toxicity of DAG by virtue of its reaction to produce PA (34). The *PAH1*-encoded PA phosphatase, which catalyzes the conversion of PA to DAG (22), counteracts DAG kinase to control the PA/DAG balance (1, 22, 30). Interestingly, the *dgk1*Δ mutation does not impart any deleterious phenotypes under typical laboratory growth conditions (1). However, the *DGK1* gene, along with its encoded DAG kinase activity, is essential for growth resumption of static cells when fatty acid synthesis is inhibited (34). In particular, DAG kinase participates in the mobilization of TAG to synthesize membrane phospholipids through PA (Fig. 1). Thus, the regulation of *DGK1* expression and DAG kinase activity is likely to influence the balance of PA and DAG, lipid metabolism, and cellular growth.

The roles of DAG kinase in lipid metabolism and cell signaling are conserved throughout evolution. In mammalian cells, however, the enzyme utilizes ATP as the phosphate donor, and its localization is cytosolic in nature (4, 6, 68–70). The mammalian enzymes associate with membranes (governed by specific interaction domains) to convert DAG to PA (4, 6, 68–70). Unlike yeast containing only one DAG kinase (1), mammalian cells possess 10 isoforms (α, β, γ, δ, ϵ, ζ, η, θ, ι, and κ) that are differentiated by their primary structures, cellular locations, and functions (4, 6, 68–70). In controlling the balance of PA and DAG, whose concentrations impact on several signaling mechanisms, the mammalian enzymes influence numerous cellular processes important to diseases such as cancer, type II diabetes, autoimmunity, and nervous system disorders (*e.g.* epilepsy) (70–75). Clearly, understanding the regulation of DAG kinase expression and activity will facilitate its control in abnormal cellular processes.

We sought to gain an understanding of the transcriptional regulation of yeast *DGK1*. Inspection of the promoter revealed that it contains the consensus sequence for interaction with the transcription factor Reb1p. Through a detailed *in vitro* analysis, we showed that Reb1p specifically binds to its recognition sequence. Moreover, the Reb1p-binding site mutations greatly diminished the expression of *DGK1 in vivo*, which was translated into reduced expressions of DAG kinase protein and activity. The consequences of losing the Reb1p-mediated activation of *DGK1* expression included the misregulation of the nuclear/ER membrane growth, and when fatty acid synthesis was inhibited, a significant defect in growth as well as in the synthesis of phospholipids from TAG mobilization. However, the residual DAG kinase activity remaining in cells expressing the Reb1p-binding site mutation supported some growth and the mobilization of TAG. Further proof that Reb1p mediated this regulation could not be obtained from the analysis of the *reb1*Δ mutant because the *REB1* gene is essential for cell growth (76).

Yeast cells resuming vegetative growth from stasis exhibit an increase in DAG kinase activity; this regulation occurs whether or not fatty acid synthesis is inhibited (34). The mechanism for this regulation was not attributed to the Reb1p-mediated activation of *DGK1* expression because the levels of P*_DGK1_-lacZ* reporter gene activity and Dgk1p did not show changes that correlated with the transient increase in DAG kinase activity. Thus, the change in DAG kinase activity appears to be regulated by a biochemical mechanism. Several phosphorylation sites have been identified in the N-terminal region of Dgk1p by phosphoproteome analyses of *S. cerevisiae* (77–81). Thus, DAG kinase activity during growth resumption from stasis might be regulated by phosphorylation/dephosphorylation. Additional work will be needed to address this hypothesis.

The essential nature of Reb1p emanates from the fact that it is required for activation of genes (*e.g. ACS1*, *ACT1*, *ENO1*, *FAS1*, *FAS2, GCY1*, *ILV1*, *PGK1*, *RAP1*, and *REB1*) involved in various aspects of cell physiology that include lipid metabolism (82–90). In particular, Reb1p interacts with the promoters of *FAS1* and *FAS2* to activate their transcription (85). Fas1p (β-subunit) and Fas2p (α-subunit) comprise the fatty-acid synthase complex (organized as α6/β6) that catalyzes a multistep process leading to the formation of fatty acids that are incorporated into lipids (91–94). Interestingly, the promoters of *ACC1* and *ACB1*, whose protein products function before and after the fatty-acid synthase reactions, also contain the Reb1p-binding site (85). Acc1p acetyl-CoA carboxylase catalyzes the conversion of acetyl-CoA to malonyl-CoA that is used by fatty acid synthase to produce fatty acyl-CoA molecules (95, 96), whereas the Acb1p acyl-CoA-binding protein delivers fatty acyl-CoA molecules into lipid biosynthetic pathways (97–99). Also, the *TGL3* promoter possesses the consensus Reb1p-binding sequence (85). Tgl3p is a major TAG lipase required for the mobilization of TAG (10, 11, 37, 39). Furthermore, the promoters of *DGA1* and *LRO1*, which encode acyltransferase enzymes responsible for the synthesis of TAG, and *CKI1* and *EKI1*, which encode kinase enzymes responsible for the synthesis of phosphatidylcholine and phosphatidylethanolamine, respectively, via the Kennedy pathway (9), contain putative sequences for Reb1p interactions. It is unknown whether Reb1p plays a role in the transcriptional activation of *ACC1*, *ACB1*, *TGL3*, *DGA1*, *LRO1*, *CKI1*, and *EKI1*. However, given their roles in lipid metabolic processes for the synthesis of TAG and its mobilization for phospholipid synthesis and growth resumption from stasis, it is reasonable to speculate that these genes might be regulated in a coordinate manner with *DGK1* by the transcription factor Reb1p. Reb1p is subject to positive and negative autoregulation (90), but whether it is regulated under these growth conditions is unknown.

In summary, this work advanced the understanding of the regulation of DAG kinase in *S. cerevisiae*. Our data supported the conclusion that *DGK1* expression was activated by the transcription factor Reb1p through its direct interaction with a Reb1p-binding site in the promoter. This study also advanced the understanding of the role that Reb1p plays in the regulation of lipid metabolism.

## Footnotes

2 The abbreviations used are:

DAG: diacylglycerol
ER: endoplasmic reticulum
PA: phosphatidate
TAG: triacylglycerol
SC: synthetic complete.
